# The effects and process of the intervention “Individual Shantala Infant Massage” in preventive child healthcare to improve parent–child interaction: study protocol for a quasi-experimental study

**DOI:** 10.1186/s12906-023-04039-z

**Published:** 2023-07-11

**Authors:** Dafna A. Windhorst, Mariska Klein Velderman, Sylvia van der Pal, Carolina de Weerth

**Affiliations:** 1 Department of Child Health, Netherlands Organization for Applied Scientific Research TNO, Leiden, the Netherlands; 2 Radboud University Medical Center, Donders Institute for Brain, Cognition and Behaviour, Nijmegen, The Netherlands

**Keywords:** Intervention, Prevention, Infant massage, Preventive Child Healthcare, Parenting, Parent–child interaction, Study design

## Abstract

**Background:**

Individual Shantala Infant Massage is an intervention that is offered by several Dutch Preventive Child Healthcare (PCH) organizations as optional preventive support, in addition to basic care as offered to all children. It targets vulnerable families and aims to enhance sensitive parenting and to reduce (effects of) parental stress. The intervention is carried out by a certified nurse. It consists of three structured home visits. Parents learn to massage their infant and receive parenting support. This study aims to investigate the effectiveness and the process of the intervention. The main hypothesis is that Individual Shantala Infant Massage leads to increased parental sensitive responsiveness, lower perceived and physiological parental stress, and improved child growth and development in the intervention group, compared to a control group where this intervention is not offered by PCH. Secondary research questions address effects on parenting confidence and parental concerns regarding the infant, the influence of background characteristics and the intervention process.

**Methods:**

The study is a quasi-experimental non-randomized trial. The aim is to include 150 infant-parent dyads in both the intervention and the control group. This takes into account possible attrition and missing data as 105 dyads with complete data per group are sufficient for analysis. All participants complete questionnaires at T0 (pre-test, child age between six-sixteen weeks), T1 (post-intervention, or ± four weeks after T0), and T2 (follow-up at five months). At T2, a hair tuft is cut from the parents’ head to measure hair cortisol levels. Data on infant growth and development is obtained from PCH files. In the intervention group, additional data is collected to evaluate the intervention process: parents complete an evaluation questionnaire at T1, nurses keep semi-structured logbooks of intervention sessions and interviews are conducted with parents and professionals.

**Discussion:**

Study results can contribute to the evidence base of infant massage as applied in Dutch PCH, and can inform parents, PCH practitioners, policy makers and researchers both inside and outside the Netherlands on feasibility and effectiveness of the infant massage intervention as applied in this format and setting.

**Trial registration:**

ISRCTN registry: ISRCTN16929184. Date (retrospectively) registered: 29/03/2022.

## Background

The basis for many opportunities and inequalities in child development and health lies in the first 1000 days of life, from conception until a child is 2 years old. This is an important phase for the physical, cognitive and socio-emotional development of children [1–4]. Parental sensitive responsiveness is an important protective factor in early childhood. A sensitive responsive parent recognizes the child's signals and knows how to respond prompt and adequately [5]. Parents who respond sensitively to the child’s signals are supporting the development of healthy emotion regulation. Moreover, they serve as a secure basis for the child from which a secure attachment relationship with the parent can be formed and from which the child can develop in a healthy manner [6–8]. Indeed, a secure attachment relationship is found to be positively related to the socio-emotional development and health of the child in later life [9–11]. In contrast, stress in early childhood or "Early Life Stress", is an important risk factor for the development of children [12, 13]. Parents who have to deal with heightened stress levels, for example due to financial, psychological or relationship problems and / or traumatic events, more often show inadequate and less sensitive parenting behaviors [14–16]. Therefore, it is precisely in the first 1000 days that infants and toddlers from vulnerable families living in stressful circumstances are at risk of falling behind in their social-emotional or cognitive development. Appropriate and early support of their parents can make a difference.

### Preventive support for vulnerable families

The Netherlands has a solid Preventive Child Healthcare (PCH) system to promote health, prevent diseases, and enable early identification of problems in the physical, psychological, social and cognitive domains in children. Basic preventive care is provided to all children aged 0–18 years, including periodic health check-ups, vaccinations, screening, and advice e.g., on safety, lifestyle, and parenting. All PCH services are provided free of charge and they have an outreach of up to 95% [17]. In addition to the basic PCH services, selective preventive care can be provided to children who grow up in disadvantaged situations, including the deployment of interventions [18]. In order to maintain the quality of interventions and to foster the implementation of effective interventions, the National Institute for Public Health and the Environment (in Dutch: Rijksinstituut voor Volksgezondheid en Milieu; RIVM) of the Ministry of Health, Welfare and Sport runs a national database of interventions. For an intervention to be registered in this database, a clear protocol and a theoretical scientific base need to be provided and be evaluated by an independent committee of experts. Depending on the amount of evidence from effect evaluations, interventions can be rated on various levels of effectiveness, ranging from ‘theoretically well-founded’ to ‘proven to be effective’ [19]. Currently, the range of interventions in this database supporting vulnerable parents to enhance positive, sensitive, responsive parenting in the first 1000 days is rather limited [20]. This holds particularly for broad, preventive, and accessible interventions.

### Individual Shantala Infant Massage

A promising accessible and preventive intervention targeting parenting and stress in vulnerable parents is “Individual Shantala Infant Massage” (in Dutch: “Shantala babymassage individueel”), which is currently registered in the intervention database as ‘theoretically well founded’ [21]. This intervention is intended for parents with a baby from 6 weeks to 9 months of age, with a risk of low sensitive parenting behavior and/or (eventually) a risk of attachment issues because of challenges or stress that parents experience, or circumstances that heighten the risk of parent–child interaction problems such as excessive infant crying. Participation in the intervention is voluntary and without costs for the parent. The infant massage intervention can be offered to all caregivers, but participants are primarily mothers. The intervention is carried out as part of PCH services by a certified nurse. During three home visits, the nurse teaches a parent how to massage their infant and simultaneously provides parenting support.

**Fig. 1:**
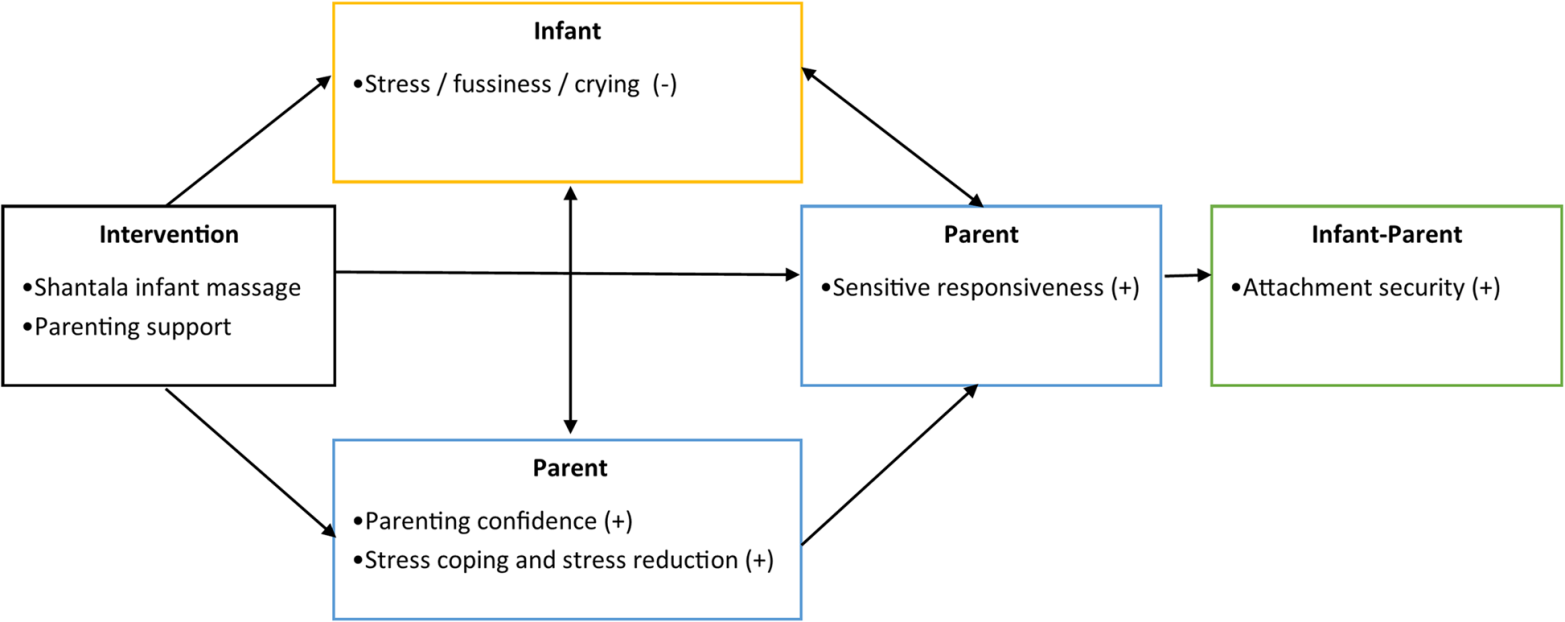
Overview of the intervention goals of Individual Shantala Infant Massage. Adapted from the intervention description of Individual Shantala Infant Massage on the website of the Dutch registry of health interventions [21]

The ultimate goal of the intervention is to foster a secure attachment relationship between parent and child by 1) increasing parental sensitive responsiveness, 2) increasing parenting confidence, and 3) reducing (effects of) stress, by improving stress coping and reducing experienced parenting stress of the parent, reducing stress and fussiness in the infant and reducing physiological stress in both the parent and the infant. The goals of the intervention are presented in Fig. 1.

### Rationale of the intervention

Effects of infant massage have been studied before, on a wide range of outcomes. In premature infants, infant massage has been associated with shorter hospital stays, better infant growth and health, as well as reduced parent anxiety, depression and stress [22, 23]). Positive outcomes have also been reported in full-term healthy babies, including growth, intestinal cramps, diarrhea, bilirubin levels, stress hormones, sleep, crying and (motor) development [24–26].

Infant massage is also supposed to promote positive parent–child interaction, because the parent learns to recognize signals better, reacts sensitively and makes eye contact e.g. [27]. Oxytocin, a hormone released in parent and child during physical contact, is thought to play an important role in this process. That is, oxytocin can counteract the physiological response to stress, reduce anxiety and increase social responsiveness, and is related to (sensitive) parenting behavior, affective touch, eye contact, communication and the emotional bond between parent and child (e.g. [25, 28–30]. Positive effects of infant massage indeed have been found on parent–child interaction (e.g., [25, 26, 31], and on attitudes about parenting, parenting competence, and parenting stress of parents [32, 33].

Despite these promising results, the evidence base is still limited. In some studies there is a high risk of bias [24, 25]. Moreover, reviews and meta-analyses point to the large variation in effect studies when it comes to techniques, form, duration, frequency and performer of infant massage, choice and rationale of outcome measures, target population and quality of research e.g. [22, 24, 25]. Consequently, results are not always unequivocal.

Research on infant massage programs to promote the parent–child relationship also showed that effects can depend on content as well as the target population [34, 35]. Based on theory, research, observations, and interviews, 14 elements were identified that are potentially important for a successful approach. Examples of these are: the facilitator (e.g. a regular professional throughout intervention sessions, who has personal and technical skills); education on infant signals, demonstration on a doll, a safe atmosphere, meeting (physical) needs of participants, social interaction, and group size. An evaluation of eight infant massage programs found large differences in the extent to which these mechanisms were applied. The conclusion of this study was that infant massage programs, if of good quality, can be used particularly effectively for parents with moderate problems [35]. Although these findings highlight the need for additional research to gain more insight into the effects of infant massage, underlying mechanisms, and the possibilities to use infant massage as an intervention, infant massage is proposed as a relatively inexpensive, theoretically plausible and easily applicable method in care [35].

Several of the elements that have been identified as important for effectiveness in infant massage interventions to improve the parent-infant interaction [34, 35] are present in the intervention Individual Shantala Infant Massage. These include: parenting support with information on infant signals, intervention provided by an experienced nurse, skilled in teaching infant massage and sensitive parenting (with a doll), offering a personalized approach, a safe environment and connection to the (physical) needs of the parent. Next to massage instructions, the intervening youth nurse provides parenting support by discussing various themes with the parent, including the pace of the infant, crying, body language and basic communication. In practice, it often proves difficult for professionals to discuss sensitivity [36]. Infant massage offers a good entry point. The parent becomes more aware of the importance of eye contact and the signals of the infant, learns how to recognize these and how to respond in a sensitive responsive manner. Because the intervention is one-on-one, it is possible to adapt to individual needs, preferences and questions of parents. It is expected that in the safe, informal atmosphere of a home visit, the parent is more likely to share questions and concerns about the child and the personal situation. Because the intervening professional is a youth nurse, she has the necessary knowledge and skills to offer parents optimal support. As a result, parents' sense of parenting competence may be enhanced, parenting stress and worries can be reduced, and parents may learn to cope better with stress. This can contribute positively to the parent–child relationship [37]. This assumption is confirmed by experiences from PCH professionals and participating parents (Meijer, D: Procesevaluatie Shantala Babymassage Individueel, CJG Rijnmond, unpublished), (Van Delft I. & Zunderman H: Procesevaluatie Shantala Babymassage Individueel, CJG Rijnmond, unpublished) and by qualitative research on a similar intervention in Norway [32].

Individual Shantala Infant Massage is offered to parents with moderate problems, who experience stress in child-raising, caring for the baby or parenting, or have an increased risk of these forms of stress or problems interacting with the baby. The embedding in PCH services facilitates initial contact with the target group. As 95% of parents of young children are in regular contact with PCH [18], professionals have insight into which families are vulnerable and provide additional support accordingly. Additionally, the format of the intervention, with the practical infant massage containing non-lingual elements with an active role for the parent, makes it accessible and deployable for a wide target group, including people from a low socioeconomic background or people with language barriers. With three home visits, it is a short-term intervention making it less intensive compared to longer interventions. Interventions with up to five sessions have been shown to be as effective in increasing parental sensitivity as interventions with five to sixteen sessions [38].

The feasibility and promise of the intervention is supported by process evaluations of a PCH organization that provides this intervention as preventive support (Meijer, D: Procesevaluatie Shantala Babymassage Individueel, CJG Rijnmond, unpublished), (Van Delft I. & Zunderman H: Procesevaluatie Shantala Babymassage Individueel, CJG Rijnmond, unpublished). PCH professionals consider infant massage a feasible intervention that is highly appreciated as a tool for helping vulnerable families and their infants. Participating parents indicated that they learned the infant massage techniques but that they also learned to better recognize the signals of their infant, felt more supported in dealing with their infant, that the contact with their infant had improved and that their infant cried less.

Taken together, the intervention Individual Shantala Infant Massage could be a promising answer to the demand for easily accessible interventions to support vulnerable families during infancy.

### Aims and hypotheses of the non-randomized controlled trial

Although the intervention is theoretically well-founded and evaluations from participating parents and professionals are promising, no controlled impact study has yet been conducted to study the effects of this intervention. Therefore, the aim of this quasi-experimental study is to investigate the effectiveness as well as the process of the Individual Shantala Infant Massage intervention as carried out in PCH.

#### Effects

Our primary research question is:

1. What are the effects of Individual Shantala Infant Massage carried out in PCH on:
  - parental stress, both perceived stress as well as physiological stress
  - parental sensitive responsiveness
  - child development and growth

We hypothesize that the intervention Individual Shantala Infant Massage will lead to an increase in parental sensitive responsiveness, lower reported and physiological stress of the parent and improved child growth and development in the intervention group, compared to a control group where the intervention is not part of the PCH care offer.

Secondary research questions are:

2. What are effects of Individual Shantala Infant massage on:
  - parenting confidence
  - (parental concerns about) crying, feeding and sleeping behaviour of the infant
3. Which subgroups may profit the most / least from the intervention?
  We hypothesize that the intervention will lead to increased parenting confidence and a decrease of parental concerns (e.g. about crying, feeding and sleeping behavior of the infant). Research question 3 is explorative.

#### Process

Finally, our last secondary research question is focused on the evaluation of the intervention process:

4. How do intermediate (professionals) and end users (parents) evaluate the intervention, including preconditions for further implementation?

## Methods

### Study design

The study has a quasi-experimental design. It is a non-randomized trial, carried out in the setting of PCH. A comparison is made between an intervention group and a control group: the intervention group consists of parent-infant dyads who receive the intervention Shantala Infant Massage as part of the care offered by their PCH organization. The control group consists of parent-infant dyads whose PCH organization does not offer this intervention as part of their standard care. Participant recruitment started in August 2021 and will end mid-2023. Data collection will continue until the end of 2023.

### Ethics

Prior to acceptance, the relevance and quality of the grant application were peer-reviewed by a team of (external) experts of the grant provider ZonMw. The Research Ethics Committee of the Radboud University Medical Centre in Nijmegen, The Netherlands (CMO region Arnhem-Nijmegen) judged this study not to be subject to the Medical Research Involving Human Subjects Act (in the Netherlands known by the Dutch abbreviation WMO, in full “Wet Medisch-wetenschappelijk Onderzoek met mensen), file number 2021–8221. The internal independent review board of TNO reviewed and approved the study’s compliance with laws and regulations concerning privacy sensitive data, reference number 2021–054. The study is conducted in accordance with guidelines and regulations of the Declaration of Helsinki. Prior to participation, all participants will receive information about the study and will provide informed consent. The study is registered in the ISRCTN registry, reference number ISRCTN16929184 [39]. Any substantial modifications to the protocol will be reported to ZonMw, the CMO, the internal review board of TNO and the ISRCTN registry.

### Participants

#### Sample size calculation

As effect sizes as found in previous research were heterogenous (see Introduction), a medium effect size (Cohen’s *d* = 0.50) was included as the basis for the power and sample size calculation. Based on an ANOVA analysis between two groups of equal size with a Cohen’s *d* effect size of 0.50, 84 participants per research arm are required for a power of 0.9. However, because it is not possible to randomly assign participants or clusters, it is necessary to correct for possible differences between the groups e.g. by adding covariates or applying propensity score weighting [40]. An effective sample size reduction of 20% is taken into account, resulting in required minimum of 105 participants per arm to retain power. In addition, we account for a non-response of 30% due to missing data and/or dropout from the study. Consequently, we aim to recruit a total of 150 participants per group; 300 participants in total.

#### Recruitment

**Fig. 2:**
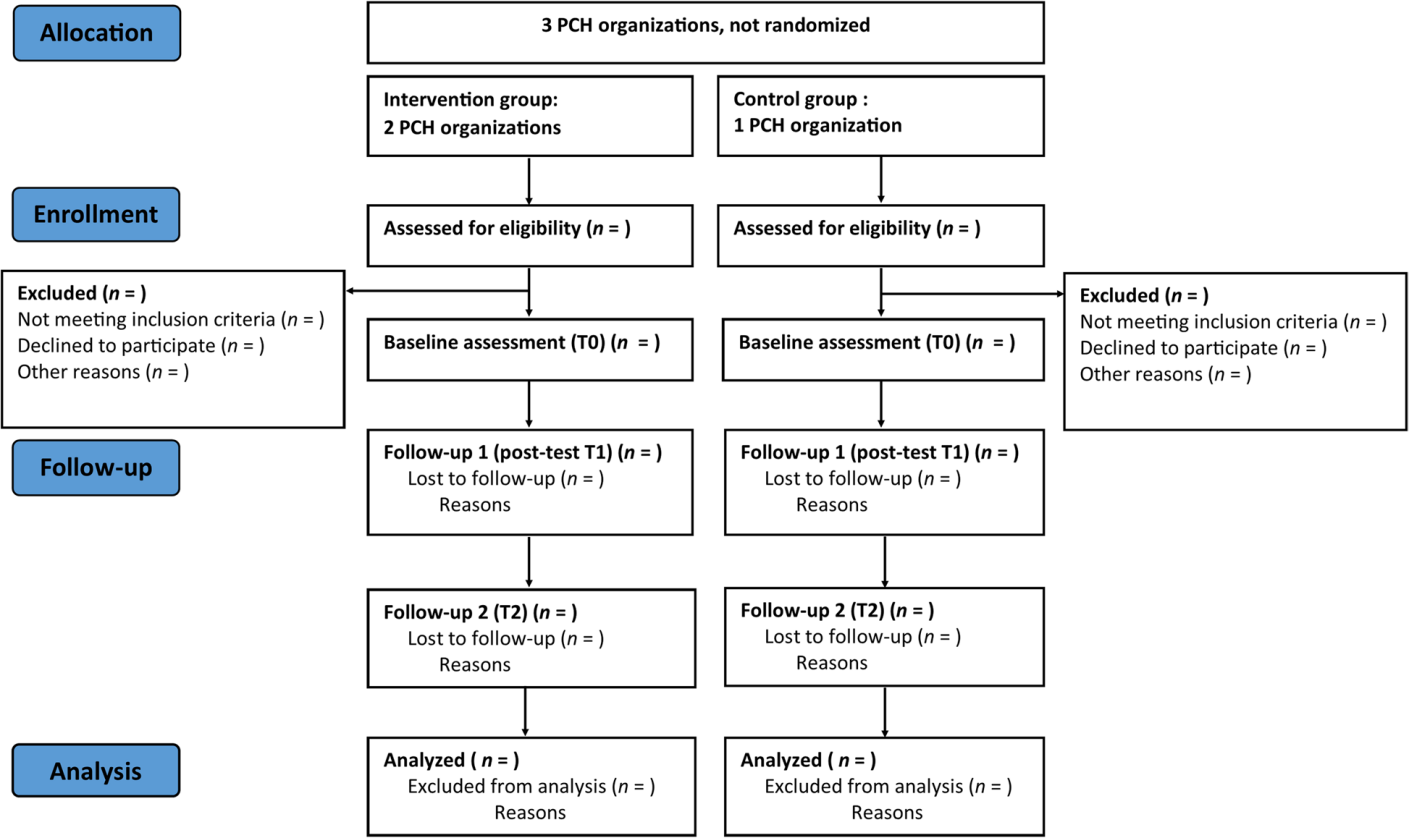
Flow chart of the non-randomized controlled trial

All participants are included via collaborating PCH organizations. The participant flow is displayed in Fig. 2.

The intervention group is recruited via two PCH organizations that both offer Shantala Infant Massage as a preventive intervention: Child and Family Centre Rijnmond (In Dutch: Centrum voor Jeugd en Gezin Rijnmond) in the city of Rotterdam and PCH Kennemerland (In Dutch: Jeugdgezondheidszorg Kennemerland) in Haarlem and surrounding (rural) areas. Parent–child dyads are eligible for participation in the study when they are about to engage in the Individual Shantala Infant Massage intervention, the infant is younger than 16 weeks and the parent is able to understand the study information and to complete the questionnaires in Dutch or English, with assistance if necessary. The parent taking part in the infant massage, is invited to participate in the study.

Participants of the control group are recruited via the PCH organization in Amsterdam, which is part of the GGD Amsterdam. Recruitment is focused on 7 PCH locations within three specific areas of the Amsterdam region with relatively high rates of vulnerability in multiple domains. Inclusion criteria for parent–child dyads regarding the age of the infant and language understanding are similar as for the intervention group. The parent who spends most time with the infant is invited to participate in the study.

For both the intervention and control group, suitable participants are notified of the study by PCH professionals. Contact information of interested parents is forwarded to the researchers with permission of the parents. Parents receive additional information on the research in a simple and accessible format. Participant information is provided in a written format, as well as in videos on a project website. To avoid desirable answers in the intervention group and to make participation more appealing for the control group, there is no emphasis on the effects of infant massage in the participant information. Instead, the focus of the study is described more generally, namely, the study is described as being about the wellbeing of infants and parents in the first 6 months and the support provided by PCH organizations during this period, including infant massage. Furthermore, parents are informed about the collection, storage, use and reuse of the data (note that in the Code of Conduct for Medical Research, ‘reuse’ is also referred to as ‘further use’), privacy regulations and rights under GDPR and contact information of the research team in case of complaints or questions.

The parents are given time (a couple of days) to consider participation. Parents’ (digital) consent form precedes the pre-test questionnaire. In case the informed consent form and the questionnaire are not yet completed after some time, the researchers contact the parents via e-mail and/or phone to check if everything was clear and as a reminder. For the intervention group, it is important that the first questionnaire is completed before the start of the intervention. All parents who provide informed consent and complete the first questionnaire are enrolled in the study. After their study participation is completed, participants receive a gift voucher of 15 euros as a thank-you.

### Intervention

Parents in the intervention group all receive Individual Shantala Infant Massage. This preventive intervention is aimed at parents with a risk of low sensitive parenting behaviour and/or (eventually) a risk of attachment issues. Examples of signals thereof taken into account by PCH at the parent level are: depressive symptoms, single, very young, unrealistic views about parenting, adverse childhood experiences, mental challenges, difficulties with parenting, pregnancy or delivery. Examples of signals in the parent–child interaction are: struggles to cope with infant crying, difficulties to adapt to the infant’s temperament, inadequate touching behaviour or little affective body contact, little to no emotional support (comforting, encouraging, supporting), lack of talking to the infant, not taking the infant into account and a lack of structure. When PCH professionals (nurses or physicians) notice these signals during PCH contact moments through observation and based on the conversation with the parent, the parent can be offered enrolment in the intervention. The intervention is offered as part of selective preventive PCH services, in addition to regular care as usual (see below).

A detailed description of the Individual Shantala Infant Massage intervention is provided on the website of the Dutch registry of health interventions [21]. The intervention consists of three weekly home visits of one hour. In exceptional cases when circumstances call for it (to the judgment of the nurse), a fourth visit is possible. The intervention is carried out at the parent’s home in a quiet and warm room. The visits have a fixed structure. Each session, the parent learns to massage a different part of the infant’s body (e.g., arms and legs). The intervening professional (i.e., a nurse) demonstrates the infant massage techniques on a doll and the parent massages the infant. The emphasis in massaging is on following the pace of the infant and being fully attentive to the infant. The intervention focuses on sensitive responses of the parent to the emotional, physical and mental state and the needs of the infant. The nurse works on increasing parental sensitivity by alerting the parent to the infant's signals (by naming and interpreting them), by showing the parent how he/she can respond positively to these signals and by naming the infant's responses to the parent’s behaviours. The nurse also informs the parent about crying, body language and basic communication as standard part of the intervention. Other topics and questions of the parent can be discussed as well, depending on his or her needs. The nurse stimulates the parent to massage the infant a couple of times a week, in between the home visits and after completion of the intervention and provides advice on how the parent can achieve this. The parent receives an information sheet depicting the various massage techniques with written instructions.

After intervention completion, the parent is allowed to contact the nurse with questions if necessary. Participation in the intervention is registered in the PCH child file, so (other) PCH professionals can ask about it during subsequent contact moments. If it appears that follow-up action and/or additional care is required for the family, necessary steps will be undertaken.

### Care as usual

All parents, both in the intervention and in the control group, receive the regular basic preventive care from their PCH organization including periodic health check-ups, the monitoring of growth and development, advice e.g. on safety, lifestyle and parenting, and possible referral to other services. Additionally, the intervention group receives the Individual Shantala Infant Massage intervention as part of selective preventive care provided by their PCH organization.

### Procedure

The study consists of three measurement moments: a pre-test (T0; at inclusion), a post-test (T1; post-intervention (intervention group) or approximately four weeks after T0 (control group), and follow-up (T2; when the child is 5 months old). Table 1 provides an overview of the various measurements at different timepoints.

**Table 1:** Overview of study measurements and timepoints

Construct	Measure	Timepoint of assessment:			
		**T0** **(pre-test)**	**T1** **(post-test)**	**T2** **(follow-up)**	**Other, namely:**
***Evaluation of intervention effects*** ***Research questions 1, 2 and 3***					
**Primary outcomes**					
Parental stress					
• Perceived stress	Questionnaire:				
	• Perceived stress Scale (PSS-10, [41])	**x**	**x**	**x**	
	•Postpartum Specific Anxiety Scale Research Short-Form (PSAS-RSF, 42)	**x**	**x**	**x**	
• Physiological stress	Hair cortisol [43, 44]				A tuft of hair of the parent will be assessed at T2. By dividing the hair tuft in segments, cortisol levels can be assessed retrospectively to compare pre- and post-intervention levels
Parental sensitive responsiveness	Questionnaire:				
	• Non-responsiveness scale (MRQ, [45])	**x**	**x**	**x**	
	• Mother-to-infant bonding scale (MIBS, [46])	**x**	**x**	**x**	
	• Questionnaire: Cry perception [47]	**x**	**x**	**x**	
Growth and development of the infant	PCH assessments: • Length • Weight • Van Wiechen scheme, [48]				Length, weight and Van Wiechen scores are assessed during each standard PCH contact moment. All data points from birth up to six months will be obtained from PCH files
**Secondary outcomes**					
Parenting confidence	Questionnaire:				
	• Karitane Parenting Confidence scale (KPCS, [49]	**x**	**x**	**x**	
Parental concerns with regard to crying feeding, sleeping of the infant	Questionnaire:				
	• Likert scales	**x**	**x**	**x**	
**Other measurements**					
Parent (and family) characteristics	Questionnaire:				
	• Date of birth	**x**			
	• Gender	**x**			
	• Country of birth of responding parent and his/her parents	**x**			
	• Educational level	**x**			
	• Relationship status	**x**			
	• Living situation (living with/without partner)	**x**			
	• Health	**x**	**x**	**x**	
	• Sleep	**x**	**x**	**x**	
	• Anxiety and depressive symptoms of the responding parent (Hospital Anxiety and Depression Scale (HADS[50, 51])	**x**			
	• Employment and working hours			**x**	
Infant characteristics	Questionnaire:				
	• Parity	**x**			
	• Single or multiple birth	**x**			
	• Gestational age	**x**			
	• Gender	**x**			
	• Birth weight	**x**			
	• Health	**x**	**x**	**x**	
Other	Questionnaire:				
	• Infant carrying	**x**	**x**	**x**	
	• Experience with infant massage	**x**	**x**	**x**	
	• (Breast)feeding			**x**	
	• Use of childcare			**x**	
	• The occurrence of stressful life events during the study period			**x**	
	• Received support from- and satisfaction with PCH			**x**	
***Evaluation of the intervention process*** ***Research question 4***					
Evaluation of the intervention by participating parents	Evaluation questionnaire (participating parents)		**x**		
Process of the intervention	Logbooks of the intervention sessions (PCH nurses)				Semi-structured logbooks will be completed after each intervention session
Intervention experiences of professionals (PCH nurses) and participating parents	Individual interviews with a number of PCH nurses and participating parents				A number of PCH nurses and parents who finished the intervention will be interviewed

The study consists of three measurement moments: a pre-test (T0; at inclusion), a post-test (T1; post-intervention (intervention group) or approximately four weeks after T0 (control group), and follow-up (T2; when the child is 5 months old)

Parents complete questionnaires at T0, T1, and T2. All questionnaires are administered digitally via a secure website by default, but participants can receive a paper version upon request. At T2, a tuft of hair is cut from the parents’ head for the assessment of hair cortisol levels. The hair is cut during the 5-month contact moment at the PCH organization or at home by a member of the research team, depending on the PCH organization. Data on infant growth and development from birth up to six months that are collected by PCH as part of standard care, will be obtained from the PCH files.

In the intervention group, additional data is collected for the purpose of process evaluation. Between T0 and T1, the nurses carrying out the intervention keep semi-structured logbooks of the intervention sessions. At T1, parents complete an extra evaluation questionnaire. Finally, interviews are conducted with a number of parents and professionals.

## Measures

### Primary outcome measures

#### Parental stress

*Perceived stress.* Perceived parental stress is measured using two self-report questionnaires: the Perceived Stress Scale (PSS-10, [41]) and the Postpartum Specific Anxiety Scale Research Short-Form (PSAS-RSF, [42]. The PSS-10 is a widely used instrument that measures the degree to which participants appraise situations as stressful and current levels of experienced stress. It consists of 10 items of a general nature. Example items are “How often have you been upset because of something that happened unexpectedly?” and “How often have you found that you could not cope with all the things that you had to do?”. For each item, participants are asked to indicate on a 5-point scale how often they felt or thought that certain way in the last month. Answer options range from 0 “never” to 4 “very often”. Total scores are calculated by reversing positively stated items and subsequently summing all items. A higher total score indicates a higher level of perceived stress. In addition to general perceived stress, maternal- and infant-focused concerns specific to the postpartum period are measured with the PSAS-RSF [42]. The PSAS-RSF is a shortened version of the PSAS, a validated 51-item instrument to measure postpartum anxiety [52]. The PSAS-RSF consists of 16 items and comprises four domains: (1) psychosocial adjustment to motherhood; (2) practical infant care anxieties; (3) maternal competence and attachment anxieties; and (4) infant safety and welfare anxieties. The measure demonstrated good construct validity and overall reliability [42]. Example items are “I have worried I will not know what to do when my baby cries” and “I have worried about my baby’s milk intake”. Participants are asked to rate each item with the answer that comes closest to how they have felt in the past 7 days, on a 4-point scale ranging from 1 “not at all” to 4 “almost always”. A total score and subscale scores can be calculated by summation of the individual item scores. Higher scores reflect higher levels of postpartum anxiety.

*Physiological stress.* Parents’ physiological stress levels are measured in hair cortisol; a reliable and valid measure for physiological stress [43, 44, 53]. The measurement of hair cortisol is a non-invasive and safe method, that provides insight in the accumulation of the cortisol stress hormone over the past months. The hair sampling is done during the 5 months contact moment (T2) at the PCH organization or at the participants’ home. About 100 hairs are cut on the back of the head, close to the scalp. The place is not visible afterwards. The tufts of hair are divided into segments of 2 cm each, enabling the comparison of stress levels before and after the intervention (i.e., pre- and posttest) between parents in the intervention and the control group. Cortisol analyses are outsourced to Dresden LabService GmbH [54].

#### Parental sensitive responsiveness

We operationalize the degree of parental sensitive responsiveness with three measures: the Non-Responsiveness Scale from the Maternal Responsiveness Questionnaire (MRQ, [45]), the Mother-to-Infant Bonding Scale (MIBS; [46]), and the Cry Perception Scale [47].

In the MRQ [45] seven contexts are presented, for example “When your baby is crying because he or she is sick or ill (e.g., has a cold, is teething, is feeling poorly after shots)” and “When your baby is crying even though he or she is well fed, well-rested, and has a fresh diaper”. For each context, different responses are described and participants are asked to rate how likely they are to respond in that specific way on a 5-point scale ranging from 1 “never” to 5 “always”. The full MRQ consists of three subscales (responsiveness, non-responsiveness, delayed responsiveness), with corresponding responses to the seven contexts. In this study, we only include the non-responsive scale with the corresponding responses: "You let your child cry for 10 min before you respond" and "You let your child cry until your child stops crying, no matter how long this takes". In combination with the seven contexts, the non-responsiveness scale consists of 13 items. Total scores of this subscale can vary between 13 and 65, with higher scores indicating less sensitive responsive behavior [45].

The MIBS [46] aims to measure feelings of parents towards their newborn babies. Parents are asked to indicate on four-point Likert scales ranging from "Very much" to "Not at all" to what extent each of the following eight feelings apply to them: “Loving, Irritated, Neutral, Happy, Dislike, Protective, Disappointed and Angry.” Total scores can vary between 0 and 24, with higher scores indicating more problematic feelings of the parent toward the infant and worse mother-to-infant bonding [46].

The Cry Perception Scale [47] measures the parents' perception of their infant's crying, using twelve 7-point bipolar rating scales. First, parents are asked in eight items how their child's cry sounds (e.g. not urgent to urgent, healthy to sick, uplifting to uncomfortable). Higher scores indicate a more negative perception of crying. Subsequently, the parent is asked to rate three items about how he / she feels when the infant is crying (e.g. not irritated to irritated, not sad at all to extremely sad). Higher scores indicate a more negative feeling. Finally, parents are asked whether their child's crying gives them the feeling of wanting to take care of the child (1 “not at all” to 7 “absolutely).

#### Child growth and development

Data on infant growth (weight and length) and development (the Van Wiechen Scheme [48, 55] from birth up to six months are obtained from files of the PCH organizations, as these data are collected as part of standard care during regular PCH contact moments.

A standard deviation score (SDS) will be calculated for weight and length for every measurement moment. This number indicates the difference with respect to the gender and age-specific average, expressed in standard deviations, enabling comparisons over time and between groups.

The Van Wiechen Scheme is the Dutch equivalent of the Bayley scales and is used to measure development in children in a systematic way [56]. It consists of developmental indicators in different domains including gross and fine motor skills, adaptation, language development, social behavior and personality. At every PCH appointment, an age-appropriate set of indicators is administered by the PCH professional [55]. These standard sets are selected in a way that about 90 percent of the children will pass. The scores on the separate Van Wiechen indicators will be used to determine a quantitative D-score (developmental score) as a continuous measure for the child’s global development. By means of the D-score it can be made clear whether a child develops according to the norms. The D-score can be used to measure differences between groups and to evaluate interventions [56].

### Secondary outcome measures

#### Parenting confidence

Parenting confidence is assessed with the Karitane Parenting Confidence Scale (KPCS, [49]). The KPCS consists of 15 items. An example item is: ‘I know what to do when my baby cries’. All items are rated on a four-point scale (0 = ’No, hardly ever’, 1 = ’No, not very often’, 2 = ’Yes, some of the time’, 3 = ’Yes, most of the time’). One item is negatively stated and therefore reverse coded. Two items can be marked as ‘Not applicable’, in that case these items are scored as a ‘2’. All items can be summed to generate a total score, which can range from 0 to 45. Higher scores indicate a higher level of parenting confidence.

#### Parental concerns with regard to the infant

Parental concerns regarding crying, nutrition and sleep behavior of the infant are assessed using three five-point Likert scales. An example item is ‘I worry about the crying of my baby’. Answer options range from ‘Strongly agree’ to ‘Strongly disagree’. On another five-point Likert scale, parents can rate if they worry about other things concerning the baby (‘Strongly agree’ to ‘Strongly disagree’), followed by an open text field where they can list things concerning the baby where they worry about.

#### Background variables

In order to control for possible group differences and to aid a correct interpretation of the results, we assess a variety of relevant background variables and possible confounders. Characteristics that are assessed with regard to the responding parent and the family include: date of birth, gender, country of birth of the responding parent and his/her parents, educational level, relationship status, living situation (living with/without partner), health, sleep, anxiety and depressive symptoms (Hospital Anxiety and Depression Scale HADS [50, 51], employment status and working hours. Child (related) characteristics that are assessed include: parity, single or multiple birth, gestational age, gender, birth weight and health. Finally, other measurements that are assessed include infant carrying, experience with infant massage, (breast)feeding, use of childcare, the occurrence of stressful life events during the study period and received support from- and satisfaction with the PCH.

#### Process variables

The process evaluation questionnaire at T1 (parents in the intervention group only) includes questions concerning frequency, duration, content of and satisfaction with the infant massage intervention. The semi-structured logbooks the intervening professionals (nurses) complete after each intervention session, consist of pre-structured registration booklets. Amongst other things, the nurses are asked to indicate to what extent the goals of the session have been achieved, if they deviated from the standard procedure and if so, why and in what way, and whether the contact and cooperation with the parents was pleasant. Finally, interviews are conducted with five to ten parents who participated in the intervention and 4–6 professionals, focused on their experiences with the intervention, recommendations, and preconditions for (further) implementation.

## Data management

All data will be handled strictly confidentially and in accordance with the guidelines of the EU General Data Protection Regulation. All procedures with regard to handling of the data are described in a Data Management Plan, which is submitted to the grant provider ZonMw.

Data used for analyses is pseudonymized after data collection, by giving each respondent a respondent number. The list of names with respondent numbers is kept separately from the research data. All digital data is stored in a secured vault at (TNO) SharePoint Online, with access restricted for project members only. The data is located within the European Union (Ireland and the Netherlands). Access to the information is only possible through Multi Factor Authentication. TNO makes a daily back-up of the information. Both for SharePoint Online and for the backup use is made of encryption 'in transit' and 'at rest’. Paper questionnaires are marked with the respondent number. After completion, the papers are scanned and subsequently destroyed. The answers are entered into the digital datasets. Both the scanned questionnaires and the datasets are stored in the secured vault at (TNO) SharePoint Online as described above. Collected hair samples are marked with the respondent number, and are securely stored in areas with limited access, both at the PCH locations and at TNO until the samples are sent away to the lab (Dresden LabService GmbH) to be analyzed and subsequently discarded.

## Statistical analysis

All data is to be handled according to standard procedures. Demographic characteristics and study outcomes will be described for each group, using means and standard deviations for continuous outcomes and proportions for categorical data. The comparability of the groups at baseline (T0) will be explored. Differences will be taken into account during further analyses. The pattern of missingness will be checked before the analyses. All measures will be controlled for outliers and checked for normality. Skewed continuous outcomes will be transformed. The level of significance (*p*-value) is set at *p* < 0.05 in all analyses.

Primary and secondary outcomes will be compared between the intervention and control groups using *T*-tests and (multilevel and multivariate) analyses of (co)variance. Differences between the intervention group and the control group at T1 and at T2 will be assessed, while correcting for the baseline measures (T0). Additionally, we will correct for possible differences between the groups by adding covariates or by applying propensity score weighting [40]. In the latter method, participants are taken more or less into account in a comparison between intervention and control groups based on covariates. Furthermore, we will investigate the influence of possible moderating factors, such as depressive symptoms of the parent and premature birth.

## Dissemination

The results of the study will be shared with relevant stakeholders in a number of deliverables. The findings will be used to update the intervention registration in the Dutch intervention database [19] and will be summarized in two factsheets. The first factsheet will be focused on the results of the effectiveness study and the other will summarize the results of the process evaluation. These deliverables will be shared with the professional users of the intervention, PCH organizations, and with policy makers, in particular municipalities. Municipalities finance the PCH and are involved in the selection of interventions that are deployed. The factsheets will be distributed via networks of implementation partners, in newsletters and via relevant websites. Examples include the national center for PCH (in Dutch: Nederlands Centrum Jeugdgezondheid) and training centers for infant massage teachers. Study results will also be translated into a practical Individual Shantala Infant Massage brochure for parents, to inform our study participants and to be used by PCH professionals to inform parents about the intervention. Relevant findings will also be published on relevant websites for parents, available for the general public. Finally, results will be shared with fellow researchers at relevant (inter)national symposia and conferences and through publications in national and international scientific journals.

## Discussion

Dutch PCH professionals have the opportunity to support vulnerable families in early life, since they are in regular contact with most parents of young children. However, there is a lack of available, easily accessible evidence-based interventions for this target group [20]. The preventive, short-term, accessible intervention Individual Shantala Infant massage, could be a promising answer to this demand.

This study protocol presents the design of a non-randomized trial to evaluate the effects and the intervention process of Individual Shantala Infant Massage in the daily practice of PCH. It is hypothesized that the intervention will lead to an increase in parental sensitive responsiveness, lower reported and physiological stress of the parent and improved child growth and development in the intervention group, compared to a control group where the intervention is not part of the standard PCH care offer. Secondary hypotheses are that the intervention will lead to increased parenting confidence and a decrease of parental concerns (e.g. about crying, feeding and sleeping behavior of the infant). An exploratory secondary research question is whether specific subgroups profit more or less from this intervention. The intervention process is evaluated based on logbooks of professionals, questionnaires for parents and interviews.

This study has several strengths. First, it is conducted in the daily practice of the PCH, which enables us to investigate the intervention in ‘real life’ rather than in an experimental setting, which supports the generalizability of our findings. Second, the study has a mixed-method design, combining information from multiple sources, including questionnaires, a physiological measurement of parental stress, PCH data on child growth and development, and an evaluation questionnaire, logbooks of the intervention sessions and interviews with both parents and professionals. However, the study also has limitations that need to be noted. The study is non-randomized, which may affect the comparability of the groups. In order to reduce the potential effects of non-randomization, we use a pre-post design. Furthermore, we attempt to control for possible group differences by assessing the most important confounders. However, the possibility of residual confounding remains and should be taken into account when interpreting the results. We also expect to encounter some challenges. The target group of the intervention, and thereby of this study, consists of parents with a young baby who may be vulnerable. This is a group where inclusion may be difficult and drop-out is to be expected. We hope to overcome this challenge by recruiting participants through and in close collaboration with PCH professionals who already are in direct contact with these parents. Furthermore, we try to lower the threshold to participate by making the study information accessible and comprehensible, by minimizing the burden by selecting questionnaires that are as short and as simple as possible, and by maintaining low-key contact with participants.

This is the first controlled effect and process evaluation of the intervention Individual Shantala Infant Massage. The study can contribute to the evidence base of individual infant massage as applied in Dutch preventive child healthcare practice. As a result, the intervention may be acknowledged at a higher level of effectiveness in the national intervention database. Study results can inform parents, PCH professionals, researchers, and policy makers, both inside and outside the Netherlands, on the feasibility and effectiveness of the Individual Shantala Infant Massage intervention. A strengthened evidence base and insights on implementation and required conditions can help to facilitate a broader implementation of this intervention by other PCH organizations in the future. This contributes to easily accessible support for vulnerable families in order to reduce (effects) of stress and to improve positive parenting and parent-infant relationships.

## Data Availability

Our project data will be accessible for further research and verification in line with the FAIR principles; a set of guiding principles in order to make data findable, accessible, interoperable and reusable [57]. The research process and the software used will be documented. Quality checks on the data will be performed to ensure that data are complete, correct and consistent. Once the project has ended and reported, a data repository for (certified) long-term archiving of our data collection will be selected, e.g. Zenodo [58]. The data will be stored for 10 years. A formal, accessible, shared, and broadly applicable language for knowledge representation will be used for the metadata. It will be ensured by the researchers that the data and their documentation will be of sufficient quality to allow other researchers to interpret and reuse them. Pseudonymized individual data will be made available on request and after the researchers’ approval (mariska.kleinvelderman@tno.nl). In the informed consent form, participants give permission for reuse of the data for studies focused on (the wellbeing of) parents and babies in the first six months. Before the data are shared, a set of terms of use will be drafted with the help of a legal advisor. For example, data will be made available on request, but may be restricted depending on whether the data has been published, purpose of usage and depending on handling fees. A Data Transfer Agreement (DTA), specifying the aim and type of data sharing, will be signed by both parties.

## Abbreviations

HADS: Hospital Anxiety and Depression Scale
KPCS: Karitane Parenting Confidence Scale
MIBS: Mother-to-infant bonding scale
MRQ: Maternal Responsiveness Questionnaire
PCH: Preventive Child Healthcare
PSAS: Postpartum Specific Anxiety Scale
PSAS-RSF: Postpartum Specific Anxiety Scale Research Short-Form
PSS-10: Perceived Stress Scale
RIVM: (In Dutch: Rijksinstituut voor Volksgezondheid en Milieu) National Institute for Public Health and the Environment
